# Supplementary material for: A biobank of pediatric patient-derived-xenograft models in cancer precision medicine trial MAPPYACTS for relapsed and refractory tumors
Source: Commun Biol. 2023 Sep 18;6:949. doi: 10.1038/s42003-023-05320-0 (PMC10507044; doi:10.1038/s42003-023-05320-0)
Supplement: Supplementary file 2 — Supplementary Information [file 42003_2023_5320_MOESM2_ESM.pdf]

Supplementary:
Supplementary Figures

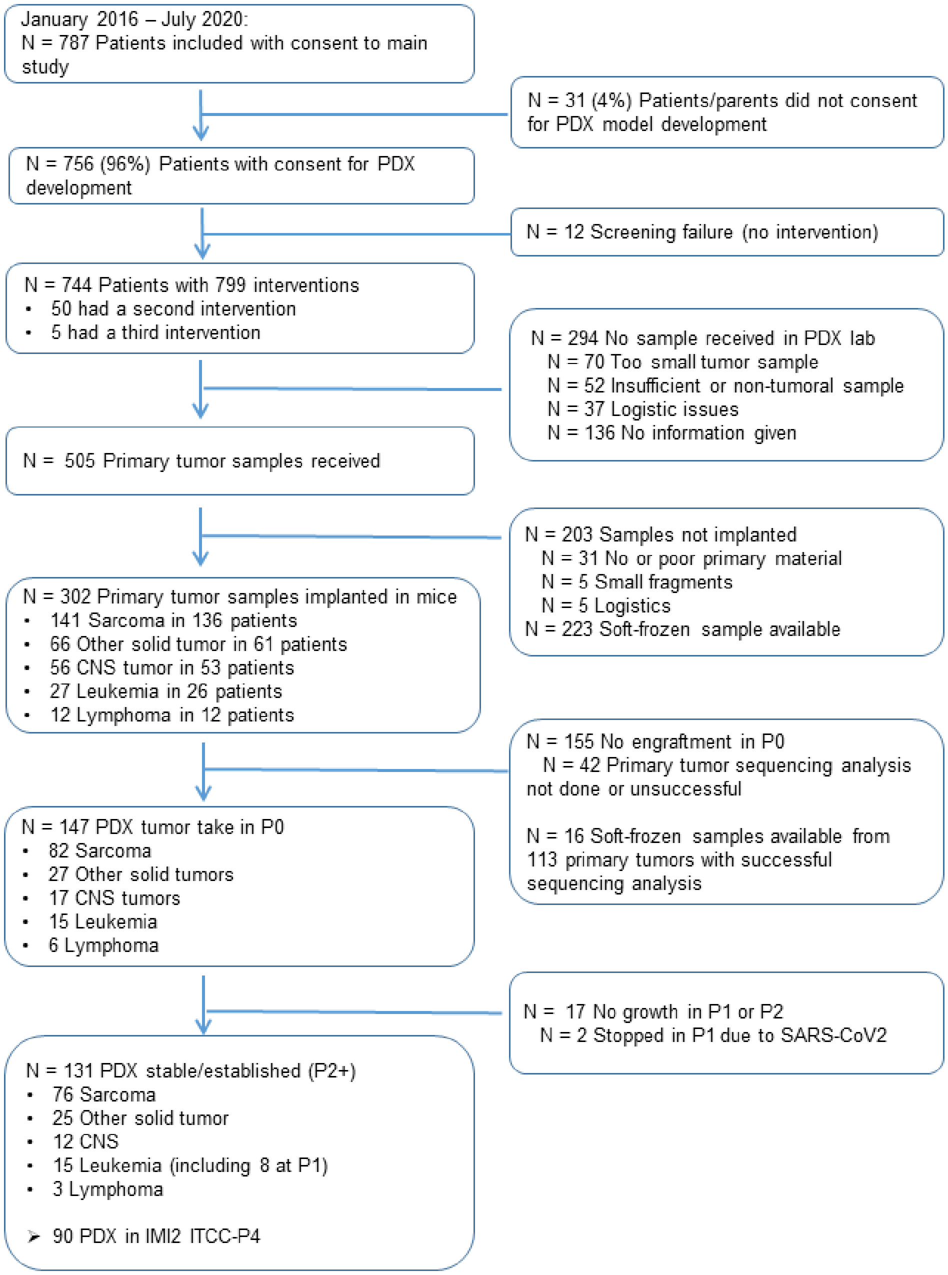

Supplementary Figure S1: Flowchart of patients and tumor samples for PDX establishment from relapsed/refractory pediatric tumors in the MAPPYACTS trial.

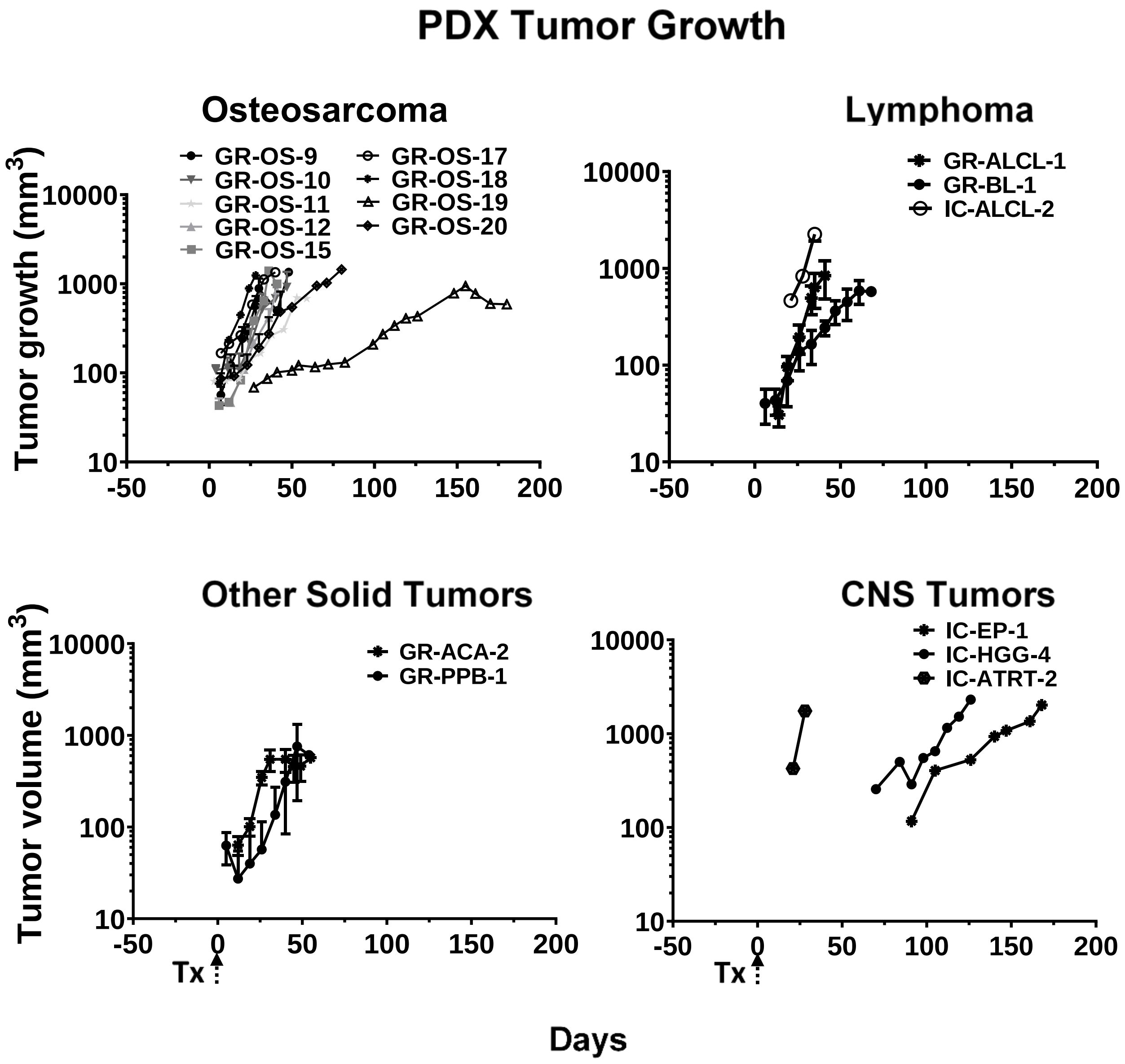

Supplementary Figure S2: Tumor growth of established subcutaneous osteosarcoma, lymphoma, other solid tumor and CNS tumor PDX. Tumor volumes depicted were obtained from 2-14 animals per PDX model; Tx: transplantation. Error bars represent ± standard error of mean (SEM).

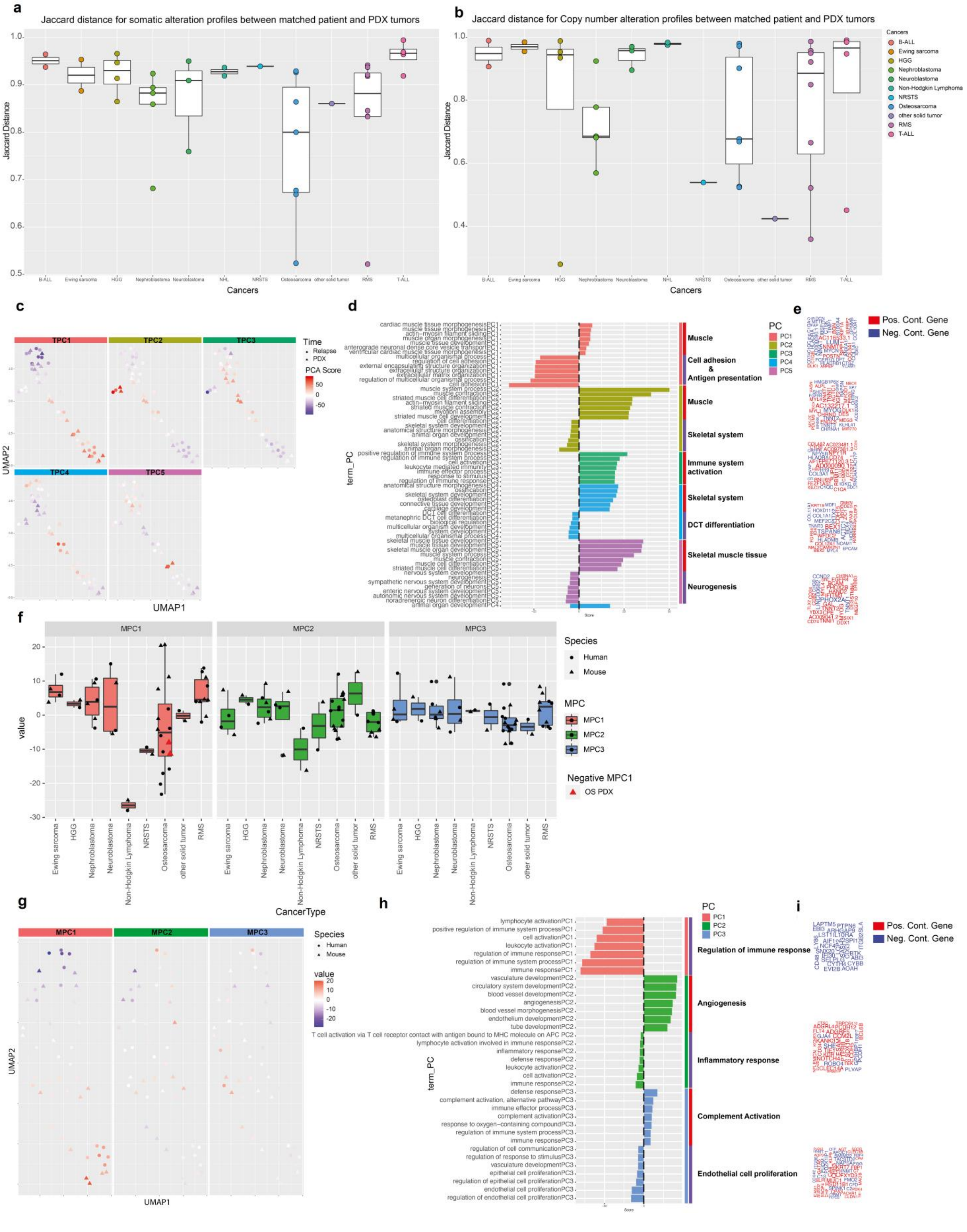
a
Jaccard distance for somatic alteration profiles between matched patient and PDX tumors
Jaccard Distance
Cancers
B-ALL
Ewing sarcoma
HGG
Nephroblastoma
Neuroblastoma
NHL
NRSTS
Osteosarcoma
other solid tumor
RMS
T-ALL
b
Jaccard distance for Copy number alteration profiles between matched patient and PDX tumors
Cancers
Non-Hodgkin Lymphoma
c
TPC1
TPC2
TPC3
TPC4
TPC5
Time
Relapse
PDX
PCA Score
UMAP2
UMAP1
d
PC
PC1
PC2
PC3
PC4
PC5
term_PC
Muscle
Cell adhesion
&
Antigen presentation
Muscle
Skeletal system
Immune system activation
Skeletal system
DCT differentiation
Skeletal muscle tissue
Neurogenesis
e
Pos. Cont. Gene
Neg. Cont. Gene
f
MPC1
MPC2
MPC3
value
Species
Human
Mouse
MPC
Negative MPC1
OS PDX
CancerType
g
MPC1
MPC2
MPC3
Species
Human
Mouse
value
UMAP2
UMAP1
h
PC
PC1
PC2
PC3
term_PC
lymphocyte activationPC1
positive regulation of immune system processPC1
cell activationPC1
leukocyte activationPC1
regulation of immune responsePC1
regulation of immune system processPC1
immune responsePC1
vasculature developmentPC2
circulatory system developmentPC2
blood vessel developmentPC2
angiogenesisPC2
blood vessel morphogenesisPC2
endothelium developmentPC2
tube developmentPC2
T cell activation via T cell receptor contact with antigen bound to MHC molecule on APC PC2
lymphocyte activation involved in immune responsePC2
inflammatory responsePC2
defense responsePC2
leukocyte activationPC2
cell activationPC2
immune responsePC2
defense responsePC3
complement activation, alternative pathwayPC3
immune effector processPC3
complement activationPC3
response to oxygen-containing compoundPC3
regulation of immune system processPC3
immune responsePC3
regulation of cell communicationPC3
regulation of response to stimulusPC3
vasculature developmentPC3
epithelial cell proliferationPC3
regulation of epithelial cell proliferationPC3
endothelial cell proliferationPC3
regulation of endothelial cell proliferationPC3
Score
Regulation of immune response
Angiogenesis
Inflammatory response
Complement Activation
Endothelial cell proliferation
i
Pos. Cont. Gene
Neg. Cont. Gene

Supplementary Figure S3: Transcriptional landscape of tumor cells and microenvironment using deconvoluted bulk RNA-Seq of matched patient/PDX samples. Jaccard distance of somatic alterations between matched patient and PDX tumors summarize for each cancer type as boxplot **(a)**. Jaccard distance of copy number alterations between matched patient and PDX tumors summarize for each cancer type as boxplot **(b)**.

Transcriptional landscape of tumor cells is summarized by five Tumor Principal Components (TPCs) **(c)** and highlighted after functional enrichment analysis the tumor cell origins for each pathology **(d)** with Word cloud illustrating the gene contributing the most to each tumor components negatively (blue) or positively (red) **(e)**. Boxplots showing the sample contributions to each MPCs and for each cancer type . Red triangles emphasize 2 osteosarcoma PDX models with contributions to MPC1 similar to osteosarcoma patient tumors **(f)**. Microenvironment Principal Components (MPCs) in patient and PDX models **(g)** suggesting a partial reconstitution of osteosarcoma microenvironment in 2 PDX, driven by the MPC1 component **(f)**, identified as the regulation of the immune response by the functional enrichment analysis **(h)** with Word cloud illustrating the gene contributing the most to each microenvironment components negatively (blue, Pos. Cont. Gene) or positively (red, Neg. Cont. Gene) **(i).** Sign of the contribution illustrates if the sample participates to the negative or positive functional enrichment fraction of the corresponding principal component.

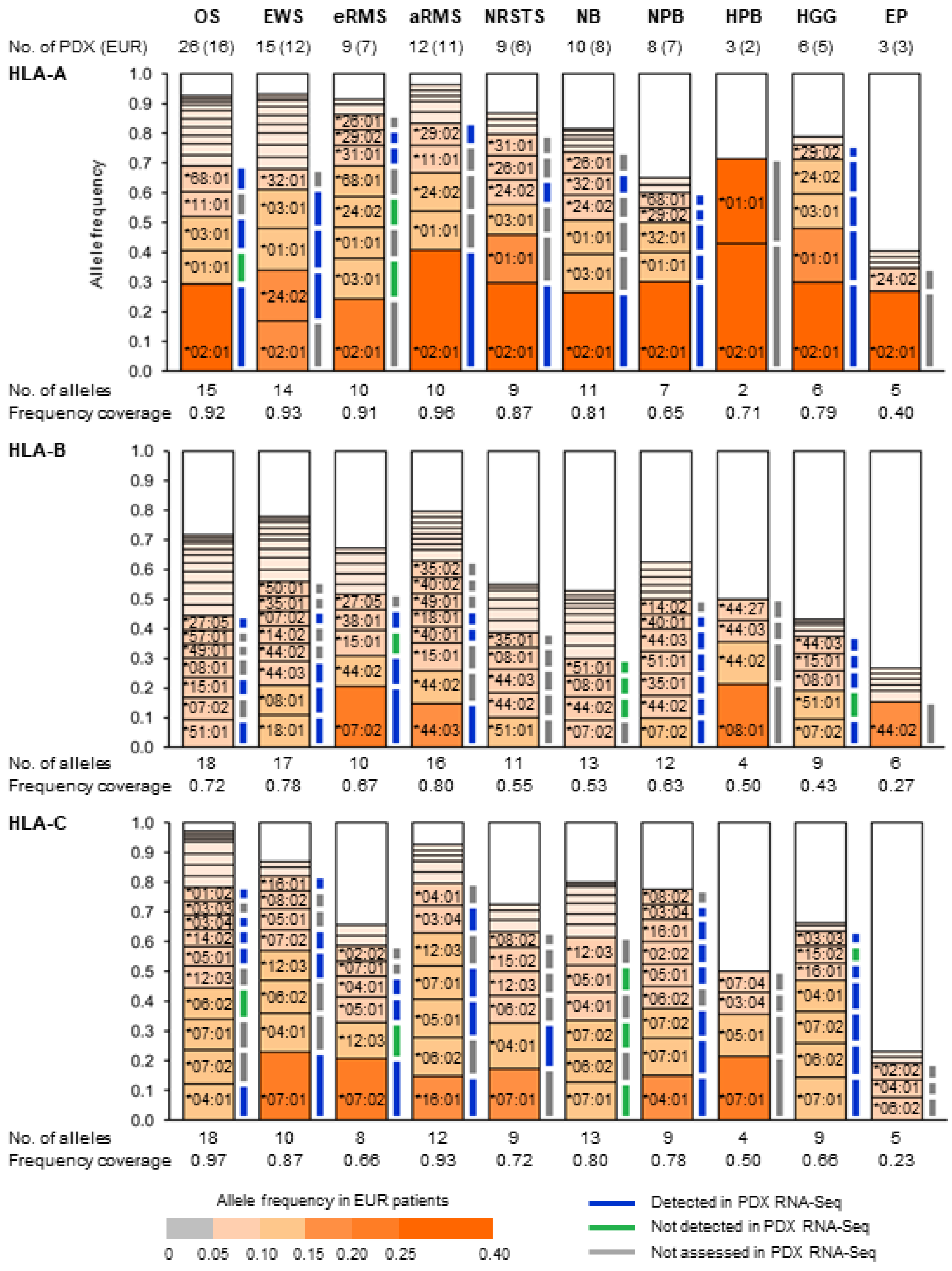

Supplementary Figure S4: HLA class I allele frequency coverage of PDX models in relation to allele frequencies in EUR patients with specific tumor types and

subtypes. HLA-A, -B and -C alleles detected in PDX models are represented by bar charts sized according to their frequency in the corresponding EUR patient cohorts, and their names are indicated for those with frequency ≥ 0.05. The total numbers of PDX models are indicated on the top, and the numbers of models established from EUR patients shown between parentheses. The numbers of alleles detected in all PDXs from a specific tumor cohort and their cumulative frequency coverage are indicated in the bottom. Blue and green bars represent supertypes with the corresponding alleles detected or not from PDX RNA-Seq, respectively, and grey bars indicate those inferred from patient NGS data.

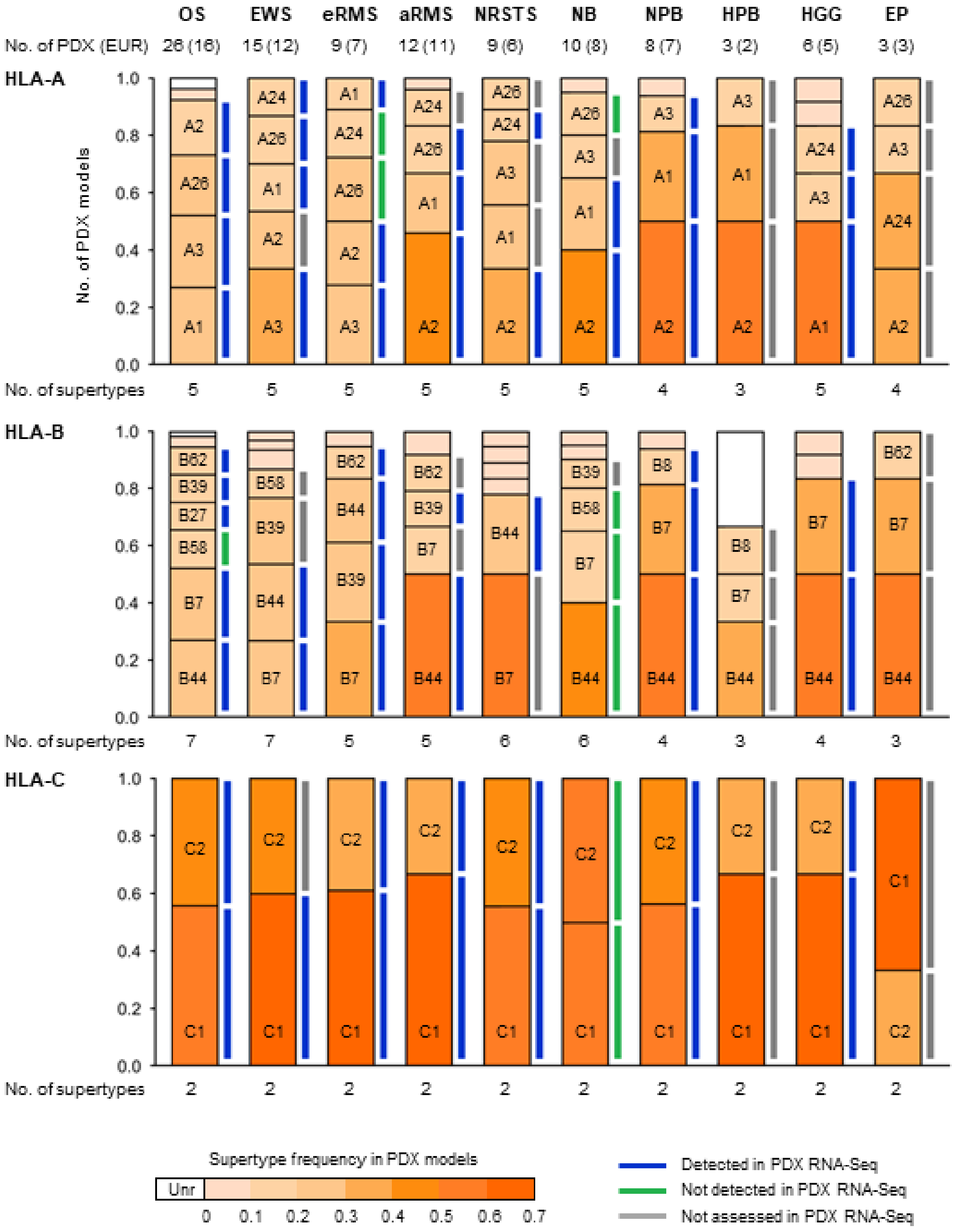

Supplementary Figure S5: HLA class I supertype frequencies in PDX models. HLA supertypes are represented by bar charts sized according to their frequencies and their

numbering indicated when frequency ≥ 0.1. The numbers of PDX models are indicated on top of the bar charts and those from EUR patients shown between parentheses. The numbers of supertypes detected in each PDX group are indicated in the bottom. Blue and green bars represent supertypes with the corresponding alleles detected or not from PDX RNA-Seq, respectively, and grey bars indicate those inferred from patient NGS data.

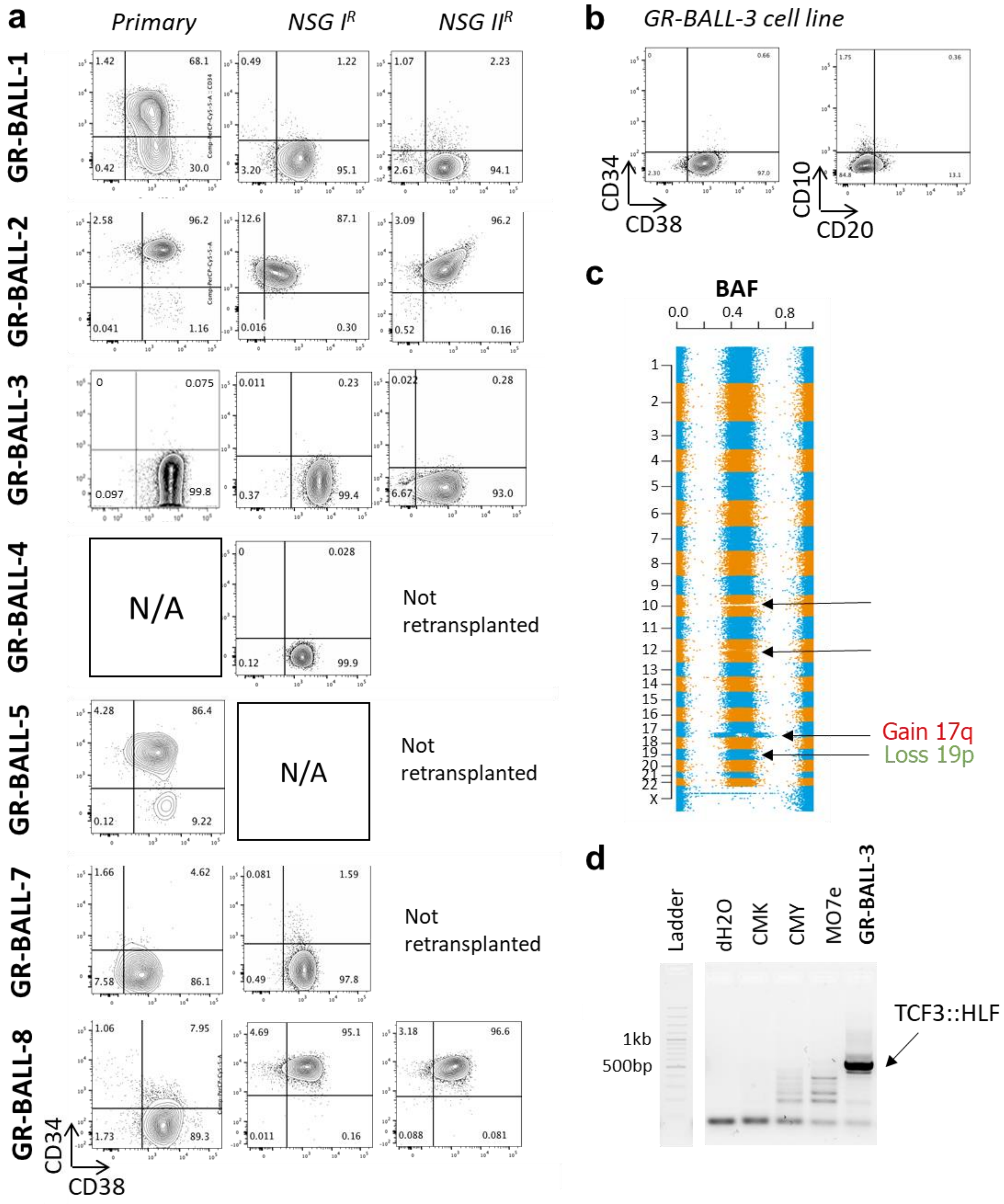
a
Primary
NSG I^R
NSG II^R
GR-BALL-1
GR-BALL-2
GR-BALL-3
GR-BALL-4
GR-BALL-5
GR-BALL-7
GR-BALL-8
N/A
Not retransplanted
N/A
Not retransplanted
Not retransplanted
CD34
CD38
b
GR-BALL-3 cell line
CD34
CD38
CD10
CD20
c
BAF
0.0
0.4
0.8
Gain 17q
Loss 19p
d
Ladder
dH2O
CMK
CMY
MO7e
GR-BALL-3
1kb
500bp
TCF3::HLF

Supplementary Figure S6: B-ALL PDX and cell line characterization. (a) Representative phenotypes of B-ALL samples engrafted into NSG recipients over 1 or 2 generations (bone marrow, gated on hCD19+ population). **(b)** Representative FAC plot assessing the phenotype of the GR-BALL-3 cell line we established (gated on hCD19+ population). **(c)** B-allele frequency (BAF, from SNP array) representation of the GR-BALL-1 genome confirming presence of gain in 17p, some segmental losses in 10p, 12q and 19p as seen in the primary sample. **(d)** PCR showing expression of the *TCF3::HLF* fusion transcript in the GR-BALL-3 cell line.

Supplementary Tables and Data

Supplementary Table S1: PDX establishment per tumor types in consenting patients in MAPPYACTS. Table summarizes in relation to main tumor types, tumor samples in patients with consent for PDX development, samples received, implanted, first tumor take rate in Passage (P) 0, established PDX models with at least 2 passages (≥P2; for some leukemia types, the model is considered established at first growth (P0*), and soft-frozen primary tumor samples still available in the laboratories. NA: not applicable, NOS non-other specified.

Diagnoses	Samples with consent N=799	Samples received n=505	Samples received from Patients n=483	Samples implanted in mice n=302	Tumor take at P0 sample n=147 (49%)	PDX established at P2 (or P0*) n=131 (43%)	Soft-frozen sample available n=223
**SARCOMAS**	**305**	**210**	**203**	**141**	**82 (58%)**	**76 (54%)**	**85**
Osteosarcoma	88	57	56	47	29 (62%)	27 (57%)	18
Ewing sarcoma	72	53	51	32	15 (47%)	15 (47%)	19
BCOR or CIC sarcoma	5	3	3	2	1 (50%)	1 (50%)	1
Other bone sarcoma	2	2	1	2	0 (0%)	0 (0%)	1
Rhabdomyosarcoma (RMS)	75	50	49	33	27 (82%)	25 (76%)	23
Alveolar RMS	35	23	23	14	12 (86%)	12 (86%)	12
Embryonal RMS	34	21	20	14	11 (79%)	9 (64%)	10
RMS NOS	6	6	6	5	4 (80%)	4 (80%)	1
Non-RMS soft tissue sarcoma (NRSTS)	63	45	43	25	10 (40%)	8 (32%)	23
Desmoplastic small round cell tumor	9	4	4	2	0 (0%)	0 (0%)	2
Malignant peripheral nerve sheath tumor	5	5	5	3	1 (33%)	1 (33%)	2
Synovial sarcoma	9	4	4	2	2 (100%)	2 (100%)	2
Undifferenciated sarcoma	8	7	6	4	2 (50%)	2 (50%)	4
Rhabdoid tumor	9	7	7	2	1 (50%)	1 (50%)	5
Other NRSTS	23	18	17	12	4 (33%)	2 (17%)	8
**OTHER SOLID TUMORS**	**188**	**118**	**110**	**66**	**27 (41%)**	**25 (38%)**	**53**
Neuroblastoma	111	71	66	38	12 (32%)	10 (26%)	33
Carcinoma	29	17	16	7	1 (14%)	1 (14%	10
Nephroblastoma	28	18	17	11	8 (73%)	8 (73%)	8
Hepatoblastoma	7	5	5	5	3 (60%)	3 (60%)	0
Other solid tumors	13	7	6	5	3 (60%)	3 (60%)	2
**Central nervous system (CNS) TUMORS**	**222**	**128**	**122**	**56**	**17 (30%)**	**12 (21%)**	**74**
High grade glioma	64	48	44	23	7 (30%)	6 (26%)	25
Low grade glioma	24	12	12	4	0 (0%)	0 (0%)	6
Medulloblastoma	50	29	28	14	4 (29%)	2 (14%)	15
Ependymoma	35	17	17	8	4 (50%)	3 (38%)	13
Atypical teratoid rhabdoid tumor	11	5	4	2	1 (50%)	1 (50%)	3
Other CNS tumors	38	17	17	5	1 (20%)	0 (0%)	12
**LEUKEMIA**	**52**	**33**	**32**	**27**	**15 (56%)**	**15 (56%)**	**7**
B-Acute lymphoblastic leukemia	18	10	10	9	7 (78%)	5 + 2* (78%)	1
T-Acute lymphoblastic leukemia	14	8	8	8	6 (75%)	6* (75%)	0
Acute myeloid leukemia	18	14	13	10	2 (20%)	2 (20%)	5
Other leukemia	2	1	1	0	na	na	1
**LYMPHOMA**	**32**	**16**	**16**	**12**	**6 (50%)**	**3 (25%)**	**4**
Hodgkin lymphoma	6	4	4	4	1 (25%)	0 (0%)	0
Anaplastic large-cell lymphoma	14	7	7	5	2 (40%)	2 (40%)	2
Other non-Hodgkin lymphoma	12	5	5	3	3 (100%)	1 (33%)	2

Supplementary Table S2: Results of the comparative histological study between primary and PDX tumors (n=51).

Tumor type	Concordant cases (n=39)			Discordant cases (n=12)
	Total number	Primary and PDX with similar features	Primary and PDX with comparable features	
Osteosarcoma	8	6	2	1
Ewing sarcoma	5	5	-	1
Rhabdomyosarcoma	10	7	3	2
Synovial sarcoma	1	1	-	0
Neuroblastoma	4	3	1	1
Nephroblastoma	2	2	-	2
CNS tumors	4	1	3	2
Lymphomas	2	2	-	0
Adenocarcinoma	1	-	1	0
Pleuropulmonary blastoma	0	-	-	1
Epithelioid sarcoma	0	-	-	1
Rhabdoid tumor	0	-	-	1
Undifferentiated sarcomas	2	2	-	0

Supplementary Table S3. HLA allele frequencies and supertypes in PDX models.

Gene	Supertype	OS (*n* = 26)	EWS (15)	eRMS (9)	aRMS (12)	NRSTS (9)	NB (10)	NPB (8)	HPB (3)	HGG (6)	EP (3)
HLA-A	A1	0,27	0,17	0,11	0,21	0,22	0,25	0,31	0,33	0,50	0,00
	A2	0,19	0,20	0,22	0,46	0,33	0,40	0,50	0,50	0,08	0,33
	A3	0,25	0,33	0,28	0,04	0,22	0,15	0,13	0,17	0,17	0,17
	A24	0,04	0,13	0,17	0,13	0,11	0,05	0,00	0,00	0,17	0,33
	A26	0,21	0,17	0,22	0,17	0,11	0,15	0,06	0,00	0,08	0,17
	Unresolved	0,04	0,00	0,00	0,00	0,00	0,00	0,00	0,00	0,00	0,00
HLA-B	B7	0,25	0,27	0,33	0,17	0,50	0,25	0,31	0,17	0,33	0,33
	B8	0,04	0,07	0,00	0,00	0,06	0,05	0,13	0,17	0,08	0,00
	B27	0,10	0,03	0,06	0,00	0,00	0,00	0,00	0,00	0,00	0,00
	B39	0,10	0,23	0,28	0,13	0,06	0,10	0,06	0,00	0,00	0,00
	B44	0,27	0,27	0,22	0,50	0,28	0,40	0,50	0,33	0,50	0,50
	B58	0,13	0,10	0,00	0,08	0,06	0,15	0,00	0,00	0,00	0,00
	B62	0,10	0,03	0,11	0,13	0,06	0,05	0,00	0,00	0,08	0,17
	Unresolved	0,02	0,00	0,00	0,00	0,00	0,00	0,00	0,33	0,00	0,00
HLA-C	C1	0,56	0,60	0,61	0,67	0,56	0,50	0,56	0,67	0,67	0,33
	C2	0,44	0,40	0,39	0,33	0,44	0,50	0,44	0,33	0,33	0,67

Supertype frequency

0 0.1 0.2 0.3 0.4 0.5 0.6 0.7
