# Supplementary material for: A biobank of pediatric patient-derived-xenograft models in cancer precision medicine trial MAPPYACTS for relapsed and refractory tumors
Source: Commun Biol. 2023 Sep 18;6:949. doi: 10.1038/s42003-023-05320-0 (PMC10507044; doi:10.1038/s42003-023-05320-0)
Supplement: Supplementary file 3 — Description of Additional Supplementary Files [file 42003_2023_5320_MOESM3_ESM.pdf]

Description of Additional Supplementary Files

File name: Supplementary Data 1
Description: Main characteristics of 131 PDX models developed in MAPPYACTS. Table presents all 131 established MAPPYACTS PDX models with the originating patient's characteristics, including primary disease, prior treatment, genetic alterations retained in the CMTB or considered of interest in the disease, and information on the established PDX models and mouse strains used. * Leukemia models considered established at the first passage; # a cell line model was established from this PDX.

File name: Supplementary Data 2
Description: HLA class I genotypes of 110 MAPPYACTS PDX models established from recurrent or refractory pediatric solid tumors inferred from PDX and patient NGS data.

File name: Supplementary Data 3
Description: Source data for the graphs and charts in the main figures.
