# Supplementary material for: A biobank of pediatric patient-derived-xenograft models in cancer precision medicine trial MAPPYACTS for relapsed and refractory tumors
Source: Commun Biol. 2023 Sep 18;6:949. doi: 10.1038/s42003-023-05320-0 (PMC10507044; doi:10.1038/s42003-023-05320-0)
Supplement: Supplementary file 7 — Reporting Summary [file 42003_2023_5320_MOESM7_ESM.pdf]

nature portfolio

Corresponding author(s): Birgit Geoerger
Manuscript COMMSBIO-23-0553-T

Last updated by author(s): 07-06-23

Reporting Summary

Nature Portfolio wishes to improve the reproducibility of the work that we publish. This form provides structure for consistency and transparency in reporting. For further information on Nature Portfolio policies, see our Editorial Policies and the Editorial Policy Checklist.

Statistics

For all statistical analyses, confirm that the following items are present in the figure legend, table legend, main text, or Methods section.

n/a	Confirmed	
☐	☒	The exact sample size (*n*) for each experimental group/condition, given as a discrete number and unit of measurement
☐	☒	A statement on whether measurements were taken from distinct samples or whether the same sample was measured repeatedly
☐	☒	The statistical test(s) used AND whether they are one- or two-sided<br>*Only common tests should be described solely by name; describe more complex techniques in the Methods section.*
☐	☒	A description of all covariates tested
☐	☒	A description of any assumptions or corrections, such as tests of normality and adjustment for multiple comparisons
☐	☒	A full description of the statistical parameters including central tendency (e.g. means) or other basic estimates (e.g. regression coefficient) AND variation (e.g. standard deviation) or associated estimates of uncertainty (e.g. confidence intervals)
☒	☐	For null hypothesis testing, the test statistic (e.g. *F*, *t*, *r*) with confidence intervals, effect sizes, degrees of freedom and *P* value noted<br>*Give P values as exact values whenever suitable.*
☒	☐	For Bayesian analysis, information on the choice of priors and Markov chain Monte Carlo settings
☒	☐	For hierarchical and complex designs, identification of the appropriate level for tests and full reporting of outcomes
☒	☐	Estimates of effect sizes (e.g. Cohen's *d*, Pearson's *r*), indicating how they were calculated

Our web collection on statistics for biologists contains articles on many of the points above.

Software and code

Policy information about availability of computer code

Data collection	In order to identify mouse reads from the PDX, reads from WES and RNAseq were first classified as of human (hg19) or mouse (mm10) origin with Xenome (v1.0). HLA typing was performed using HLA-HD from WES and RNA-Seq, and using both HLA-HD and HLAProfiler from RNA-Seq. When both algorithm identified a discordant genotype, HLA typing was repeated using Optitype, xHLA and HISAT-genotype, from patient normal and PDX WES. A final consensus genotype was deduced when two algorithms identified the same allele(s) from two different NGS samples. Metabolic function analysis was performed using Cellfie algorithm on the RNA-Seq of primary tumors and of the human fraction of the PDXs.
Data analysis	All WES reads classified as human were then aligned using BWA (0.7.12; BWA, RRID:SCR_010910). The variant calling was performed using Varscan2 (2.3.9; VARSCAN, RRID:SCR_006849). The copy number calling was performed using Sequenza (2.1.2). Quantification of gene expression from the RNA-Seq human fraction was estimated using Salmon (0.9.0) on the GENCODE reference transcriptome (v27). Gene fusion calling was performed using the nf-core rnafusion pipeline running Arriba, Star-Fusion, EricScript and Squid. The differential gene expression analysis was performed using the R package Deseq2 (DESeq2, RRID:SCR_015687). HLA allele frequencies were calculated for patients with a European (EUR) ancestry fraction of at least 70%, determined using the EthSEQ pipeline. Metabolic analysis was produced using Recon version 2.2 as reference metabolic model network.

For manuscripts utilizing custom algorithms or software that are central to the research but not yet described in published literature, software must be made available to editors and reviewers. We strongly encourage code deposition in a community repository (e.g. GitHub). See the Nature Portfolio guidelines for submitting code & software for further information.

Data

Policy information about availability of data

All manuscripts must include a data availability statement. This statement should provide the following information, where applicable:

- Accession codes, unique identifiers, or web links for publicly available datasets
- A description of any restrictions on data availability
- For clinical datasets or third party data, please ensure that the statement adheres to our policy

Data availability:
All relevant data generated or analyzed for this study are available within the article and its Supplementary Information files or from the corresponding author upon reasonable request. Sequencing data and basic clinical annotations from all patients and PDX have been deposited in European Genome-phenome Archive (EGA; hosted by the EBI and CRG) with the data set accession code EGAS00001005935 and EGAS00001007327 respectively. Further information about EGA can be found on https://ega-archive.org ("The European Genome-phenome Archive of human data consented for biomedical research"; http://www.nature.com/ng/journal/v47/n7/full/ng.3312.html). Source data for the graphs and charts in the main figures is available as Supplementary Data 3. The analysis codes are available on a public GitHub server.

Human research participants

Policy information about studies involving human research participants and Sex and Gender in Research.

Reporting on sex and gender	This information is described in the supplementary Table S2 that details the characteristics of all 131 PDX models and their originating patient tumor with origin, including gender of the originating patients.
Population characteristics	The trial MAPPYACTS recruited 787 pediatric patients; 756 (96%) patients and their parents consented to the optional ancillary study of preclinical model development. 302 tumor samples were transplanted in animals for PDX development. 131 PDX models were obtained from patients with recurrent or refractory pediatric cancer with 0.5-30.8 years old, 113 were solid tumors (76 sarcoma, 25 other non-central nervous system (CNS) tumors, 12 CNS tumors), 3 lymphomas and 15 leukemias presenting with more than 50% leukemic blasts. Supplementary Table S2 details the characteristics of all 131 PDX models and their originating patient tumor with origin, prior therapies, molecular alterations retained in the clinical molecular tumor board that were considered as actionable, of interest or disease specific.
Recruitment	Patients with recurrent or refractory pediatric cancer underwent following informed consent a tumor biopsy, surgical resection, blood or bone marrow sampling for molecular characterization within the MAPPYACTS trial 2 in 18 medical centers in France, Ireland, Italy and Spain. Main inclusion criteria were age below 18 years at diagnosis, refractory or relapse, evaluable or measure disease at inclusion, good clinical performance status and life expectancy more than 3 months, no organ toxicity more than grade 1 and potential eligible to an early clinical trial. The development of preclinical tumor models was optional and performed only for the patients with specific consent and only if sufficient material was available.
Ethics oversight	The MAPPYACTS trial (NCT02613962) was approved by independent ethics committees and national medical authorities in France, Italy, Spain, Ireland, and conducted according to the principles of the Declaration of Helsinki.<br>The development of preclinical tumor models was optional and performed only for the patients with specific consent and only if sufficient material was available.

Note that full information on the approval of the study protocol must also be provided in the manuscript.

Field-specific reporting

Please select the one below that is the best fit for your research. If you are not sure, read the appropriate sections before making your selection.

☒ Life sciences ☐ Behavioural & social sciences ☐ Ecological, evolutionary & environmental sciences

For a reference copy of the document with all sections, see nature.com/documents/nr-reporting-summary-flat.pdf

Life sciences study design

All studies must disclose on these points even when the disclosure is negative.

Sample size	One hundred thirty-one PDX models were obtained from patients with recurrent or refractory pediatric cancer, 113 were solid tumors (76 sarcoma, 25 other non-central nervous system (CNS) tumors, 12 CNS tumors), 3 lymphomas and 15 leukemias. No sample size calculation was performed (not aplicable in this study). Patients with recurrent or refractory pediatric cancer underwent following informed consent a tumor biopsy, surgical resection, blood or bone marrow sampling for molecular characterization within the MAPPYACTS trial 2 in 18 medical centers in France, Ireland, Italy and Spain. Main inclusion criteria were age below 18 years at diagnosis, refractory or relapse, evaluable or measure disease at inclusion, good clinical performance status and life expectancy more than 3 months, no organ toxicity more than grade 1 and potential eligible to an early clinical trial. The development of preclinical tumor models was optional and performed only for the patients with specific consent and only if sufficient material was available.

Data exclusions	No data were excluded.
Replication	PDX development was performed in at least 2 mice for growth curves; Whole exome and RNA sequencing and proteomics were performed in each PDX and analyzed as tumor type cohorts. No replicates were performed
Randomization	Not aplicable in this study. the objective was the PDX development.
Blinding	Not aplicable in this study. the objective was the PDX development.

Reporting for specific materials, systems and methods

We require information from authors about some types of materials, experimental systems and methods used in many studies. Here, indicate whether each material, system or method listed is relevant to your study. If you are not sure if a list item applies to your research, read the appropriate section before selecting a response.

Materials & experimental systems

n/a	Involved in the study
☐	☒ Antibodies
☒	☐ Eukaryotic cell lines
☒	☐ Palaeontology and archaeology
☐	☒ Animals and other organisms
☐	☒ Clinical data
☒	☐ Dual use research of concern

Methods

n/a	Involved in the study
☒	☐ ChIP-seq
☒	☐ Flow cytometry
☒	☐ MRI-based neuroimaging

Antibodies

Antibodies used	mouse anti-human Ki67h antibody (1:20; Dako), mouse anti-human CD3 (1:20; Dako), CD20 antibodies (1:200; Dako)
Validation	antibodies were used according to manufactorer's guidelines; positive and negative controls were included

Animals and other research organisms

Policy information about studies involving animals; ARRIVE guidelines recommended for reporting animal research, and Sex and Gender in Research

Laboratory animals	Heterotopic and/or orthotopic PDX were established depending on the tumor size and tumor histology in 3-7 weeks immunocompromised female and male Swiss athymic Nude (Crl:NU(Ico)-Foxn1nu), SCID (CB17/Icr-Prkdcscid/IcrIcoCrl), NSG (NOD.Cg-PrkdcscidIL2rgtm1Wjl/SzJ) or NSG expressing human cytokines (NSG-IL) mice, obtained from the institutional animal facilities or from Charles River animal facilities, as reported previously. Animals were housed in each respectively institution.
Wild animals	No wild animals used.
Reporting on sex	Heterotopic and/or orthotopic PDX were established depending on the tumor size and tumor histology in 3-7 weeks immunocompromised female and male Swiss athymic Nude (Crl:NU(Ico)-Foxn1nu), SCID (CB17/Icr-Prkdcscid/IcrIcoCrl), NSG (NOD.Cg-PrkdcscidIL2rgtm1Wjl/SzJ) or NSG expressing human cytokines (NSG-IL) mice, obtained from the institutional animal facilities or from Charles River animal facilities.
Field-collected samples	Not applicable
Ethics oversight	Animal care and use were performed in accordance with international guidelines and the recommendations of the European Community (2010/63/UE). Experimental procedures were specifically approved by the ethics committee, the France Ministry of Agriculture or Italian Ministry of Health; Gustave Roussy CEEA26 (CEEA PdL N°6, approval number: 2015032614359689 V7, 1281.01, C75-05-18, 2012-017), Institut Curie CEEA-IC #118 (APAFIS#11206-2017090816044613-v2), Centre Léon Bérard CEEA CECCAPP N°15, (APAFIS#10079), CEA (APAFIS#9458-2017033110277117 v2), Nantes University (APAFIS#32043-2021061811307790 v2), University Strasbourg (APAFIS #2017021410378167), Fondazione IRCCS Istituto Nazionale dei Tumori (OPBA authorization: INT 03_2018, Italian Ministry of Health authorization: 646/2018-PR), IDIBELL animal facility committee (AAALAC Unit1155).

Note that full information on the approval of the study protocol must also be provided in the manuscript.

Clinical data

Policy information about clinical studies

All manuscripts should comply with the ICMJEguidelines for publication of clinical research and a completedCONSORT checklist must be included with all submissions.

Clinical trial registration	MAPPYACTS; ClinicalTrials.gov identifier: NCT02613962

Study protocol	Patient data study published in Berlenga at al. Cancer Discov 2022;12:1266–81; doi: 10.1158/2159-8290.CD-21-1136
Data collection	Patients data were collected in electronic CRFs and published in Berlenga at al. Cancer Discov 2022;12:1266–81; doi: 10.1158/2159-8290.CD-21-1136
Outcomes	The PDX study was exploratory and results on the establishment rates are included in the present manuscript
